# Whole-genome sequencing of antimicrobial-resistant *Salmonella enterica* isolates from a *Cairina moschata* carcass

**DOI:** 10.1016/j.dib.2023.108932

**Published:** 2023-01-25

**Authors:** Trung Thanh Nguyen, Hoa Vinh Le, Da Pham Xuan, Trung Nghia Vu, Minh Hong Nguyen, Huyen Thi Thanh Tran

**Affiliations:** a Department of Food Microbiology and Genetically Modified Food, Vietnam National Institute for Food Control, Cau Giay, Hanoi, Vietnam; b Center for Genetic and Reproductive Health, Faculty of Medicine - Vietnam National University, Ho Chi Minh City, Vietnam; c Department of Medical Epidemiology and Biostatistics, Karolinska Institutet, Solna, Stockholm, Sweden; d Bioresource Research Center, Phenikaa University, Hanoi 12116, Vietnam; e Vinmec Research Institute of Stemcell and Gene Technology, Hai Ba Trung, Hanoi, Vietnam

**Keywords:** *Salmonella enterica*, Whole-genome sequencing, Antibiotic resistance, Cairina moschata

## Abstract

*Salmonella enterica* is one of the most common agents of foodborne bacterial illness with poultry being an important reservoir. The indiscriminate use of antimicrobial compounds in poultry farming increasingly leads to antimicrobial-resistant (AMR) which threatens the health of both animals and humans. Antimicrobial-resistant *Salmonella enterica* from the poultry can spread to human through the direct contact with infected poultry or fecal contaminated environments. Antimicrobial-resistant *S. enterica,* especially fluoroquinolone-resistant nontyphoidal *Salmonella* is in the list of global health concern stated by the World Health Organization (WHO). Here we report the whole-genome sequencing data and de novo genome assemble of antimicrobial-resistant *S. enterica* strains S8 and S9 from the *C. moschata* carcass collected in Vietnam. Genomic DNA of *S. enterica* were extracted and subjected to whole-genome sequencing using Illumina MiSeq platform. The genome size of antimicrobial-resistant *S. enterica* strain S8 is 4,707,459 bp with a GC-content of 52.38%, containing 10 antimicrobial resistant genes. The genome size of antimicrobial-resistant *Samonella enterica* strain S9 is 4,923,944 bp with a GC-content of 52,39%, containing 10 antimicrobial resistance genes. Our data provided the insights on antimicrobial resistant genes of *S. enterica* isolates from the *C. moschata* carcass, which help to understand the infection mechanism of antimicrobial-resistant *S. enterica* in human.

**Specifications Table**

**Table:** 

Subject	Microbiology
Specific subject area	Bacteriology, genomics
Type of data	Whole-genome sequence data in FASTA format, tables.
How the data were acquired	Whole-genome sequencing with an Illumina MiSeq system
Data format	Tables Raw sequencing reads (fastq) *de novo* assembled sequences (Fasta), (fastq)
Description of data collection	Genomic DNA was isolated from pure cultures of antimicrobial-resistant *S. enterica* S8 and S9 isolates from *C. moschata* carcasses and sequenced by Illumina MiSeq
Data source location	*Salmonella enterica* S8 and S9 strains were isolated from the *C. moschata* carcasses purchased at wet markets (21,01 N 105,48 E and 20,59 N 105,48 E, respectively) in Hanoi, Vietnam
Data accessibility	The sequencing data was deposited at the National Center for Biotechnology Information sequence Read Archive database with accession numbers of SRR21051812 (S8) and SRR21051811 (S9). The deposited whole-genome sequencing data can be accessed at https://www.ncbi.nlm.nih.gov/sra/SRX17067005 https://www.ncbi.nlm.nih.gov/sra/SRX17067006

## Value of the Data

• The data provides the information of whole genome sequence of antimicrobial-resistant *Salmonella enterica* S8 and S9 isolates from the *C. moschata* carcass collected in Vietnam.
• Whole-genome sequence data of antimicrobial-resistant *S. enterica* provides insights on antimicrobial resistance genes in *S. enterica genome* The data enables further understanding of the mechanism and treatment of diseases caused by antimicrobial-resistant *S. enterica* in human.
• Whole genome sequence data are available in the gene bank for the biomedical community and can be applied to develop the effective test kits based on the information of antimicrobial resistance genes.

## 1 Objective

Among *Salmonella* genus, *Salmonella enterica* is the common factor that causes foodborne outbreaks. *Salmonella enterica* consists of 6 subspecies, which compose of more than 2600 serovars. *S. enterica* subsp. *enterica* is responsible for more than 99% of human salmonellosis globally. In Vietnam, due to the expansion in antimicrobial resistance of pathogenic bacteria and increased availability of whole-genome sequencing tools, publications of whole-genome sequencing data of pathogenic *Salmonella* in Vietnam have risen in recent years [1], [2], [3], [4], [5], [6]. Here we isolated the antimicrobial-resistant *Salmonella* S8 and S9 isolates from *Cairina moschata* carcasses (commonly called Muscovy duck) carcasses collected from several wet markets in Hanoi, Vietnam. The *Salmonella* S8 and S9 isolates were classified as *Salmonella enterica* serovar Muenster and serovar Infantis, respectively. Both antimicrobial-resistant *S. enterica* S8 and S9 isolates have not been published in databases related to pathogenic bacteria in Vietnam. Thus, in this study, we analyzed the whole genome sequencing of *S. enterica* serovar Muenster S8 and *S. enterica* serovar Infantis S9 strains to give insights into antimicrobial resistance genes of *Samonella* isolates from *C. moschata* carcasses.

## 2 Data Description

Here we reported the whole genome sequencing of *S. enterica* serovar Muenster S8 and *S. enterica* serovar Infantis S9 together with antimicrobial resistant genes.

The assembled genome size of antimicrobial-resistant *S. enterica* S8 strain was 4,707,459 bp with a GC content of 52,38%. While antimicrobial-resistant *S. enterica* strain S9 accounted for a genome size of 4,923,944 bp and a GC content of 52,39% (Table 1). The genomes dataset of strain S8 and S9 were assigned as ST-321 and ST-32 in the public databases for molecular typing and microbial genome diversity (PubMLST), respectively. The screening results for contigs of antimicrobial resistant genes suggested that both genomes of *S. enterica* S8 and S9 strains contained a number of antimicrobial resistant genes against multiple antibiotics, including tetracycline, quinolone, gentamicin, beta-lactam, streptomycin, extended-spectrum beta-lactamases (ESBLs). Mass screening for contigs of antimicrobial resistant or virulence genes (ABRicate) identified all the antibiotic resistant genes in Tables 2 and 3.

**Table 1:** General genome characteristics of *Salmonella* isolates.

*Salmonella* isolate	Species identified	Multi-Locus Sequence Typing (MLST)	Raw read numbers	Number of Contigs (after assembly)	Total GC Content	Total Length (bp)
SRR21051811 (S8)	*Salmonella enterica* subsp. *enterica* serovar Schwarzengrund str. CVM19633	ST-321	688,022	383	52.38%	4,707,459
SRR21051812 (S9)	*Salmonella enterica* subsp. *enterica* serovar Heidelberg str. 41578	ST-32	710,700	443	52.39%	4,923,944

**Table 2:** ABRicate screening of contigs for antimicrobial resistance and virulence genes of *Salmonella* strain S8.

Sequence	Start	End	Gene	%Coverage	%Identity	Accession	Resistance
contig00062	10476	11267	*sul3*	100.00	100.00	NG_048120.1	Sulfonamide
contig00201	4168	5367	*tet(A)*	100.00	99.92	NG_048154.1	Tetracycline
contig00201	6713	7549	*aph(6)-Id*	100.00	100.00	NG_047464.1	Streptomycin
contig00350	3727	4383	*qnrS1*	100.00	100.00	NG_050543.1	Quinolone
contig00417	392	1252	*aac(3)-IId*	100.00	100.00	NG_047251.1	Gentamicin
contig00477	2139	2996	*bla*_LAP-2_	100.00	100.00	NG_049264.1	Beta-lactam
contig00610	1303	2178	*bla*_CTX-M-55_	100.00	100.00	NG_049006.1	Cephalosporin
contig00618	373	846	*dfrA14*	100.00	100.00	NG_056035.1	Trimethoprim
contig00618	957	1409	*arr-2*	100.00	100.00	NG_048580.1	Rifamycin
contig00695	216	1037	*lnu(F)*	100.00	99.88	NG_047927.1	Lincosamide

**Table 3:** ABRicate screening of contigs for antimicrobial resistance genes of *Salmonella* strain S9.

Sequence	Start	End	Gene	%Coverage	%Identity	Accession	Resistance
contig00152	7743	8942	*tet(A)*	100.00	100.00	NG_048154.1	Tetracycline
contig00209	354	1193	*sul1*	100.00	100.00	NG_048082.1	Sulfonamide
contig00209	6910	7749	*sul1*	100.00	100.00	NG_048082.1	Sulfonamide
contig00288	3985	5010	*aph(4)-Ia*	100.00	100.00	NG_047456.1	Hygromycin
contig00288	5239	6015	*aac(3)-IVa*	100.00	100.00	NG_047253.1	Gentamicin
contig00319	423	1298	*bla*_CTX-M-65_	100.00	100.00	NG_049016.1	Cephalosporin
contig00319	4581	5456	*bla*_CTX-M-65_	100.00	100.00	NG_049016.1	Cephalosporin
contig00354	2371	2844	*dfrA14*	100.00	100.00	NG_047696.1	Trimethoprim
contig00616	270	1061	*aadA1*	100.00	100.00	NG_047324.1	Streptomycin
contig00824	221	1036	*aph(3′)-Ia*	100.00	100.00	NG_047431.1	Kanamycin

## 3 Experimental Design, Materials and Methods

### 3.1 Genomic DNA extraction and sequencing

*S. enterica* S8 and S9 strains were cultured in Brain Heart Ìnfusion broth (BHI broth, Merck, Germany) at 37°C for 18 – 24 hours. 1mL of overnight culture was centrifuged at 2,000 rpm for 10 minutes at room temperature and harversted cells were applied for genomic DNA extraction. The DNA extraction procedure was executed by using PureLink™ Genomic DNA Mini Kit (Invitrogen, Thermofisher scientific) according to the manufacturer's protocol. A library was prepared for sequencing using Nextera XT DNA Library Preparation Kit (Nextera XT, Illumina, San Diego, CA, USA) and WGS sequencing was performed using the Illumina MiSeq system (Illumina, San Diego, CA, USA), as described by the respective manufacturers.

### 3.2 Genome assembly, annotation and comparative genomic analysis

Read trimming was carried out using Trimmomatic removing the sequence adaptor [7]. Quality control was conducted with FastQC (https://www.bioinformatics.babra-ham.ac.uk/projects/fastqc/). *De novo* assembly was performed using SPAdes 3.15 [8]. Contigs were ordered against a *Salmonella enterica* sbsps*. enterica* serovar Typhimurium strain ATCC 14028 using ABACAS v1.3.1 with -dmbc setting [9]. Whole genome sequencing data of both isolates were then identified by using Serotype (SISTR1 + SeqSero2) web tool from the EnteroBase website (http://enterobase.warwick.ac.uk/), the database used for analysis is based on *Salmonella In Silico* Typing Resource (SISTR) platform [10]. Abricate was applied for screening of the antibiotic resistant genes, plasmid replicons and virulence genes [11]. The antibiotic resistant genes were identified by screening of the draft genome against NCBI ARM finderPlus databases [12]. The search of plasmid replicons was conducted by screening of the draft genome against PlasmidFinder database [13]. The virulence genes were detected by screening of the draft genome against Virulence Factors Data Base [14].

## Ethics Statements

This work did not involve studies with animals and humans.

## CRediT authorship contribution statement

**Trung Thanh Nguyen:** Conceptualization, Methodology, Software, Writing – original draft. **Hoa Vinh Le:** Investigation, Visualization. **Da Pham Xuan:** Conceptualization, Methodology. **Trung Nghia Vu:** Software, Validation. **Minh Hong Nguyen:** Writing – review & editing. **Huyen Thi Thanh Tran:** Validation, Supervision.

## Declaration of Competing Interest

The authors declare that they have no known competing financial interests or personal relationships that could have appeared to influence the work reported in this paper.

## Data Availability

Whole genome sequencing of Salmonella enterica S9 strain (Original data) (Mendeley Data) Whole genome sequencing of Salmonella enterica S8 strain (Original data) (Mendeley Data)
